# Toward Sensor-Based Context Aware Systems

**DOI:** 10.3390/s120100632

**Published:** 2012-01-09

**Authors:** Yoshitaka Sakurai, Kouhei Takada, Marco Anisetti, Valerio Bellandi, Paolo Ceravolo, Ernesto Damiani, Setsuo Tsuruta

**Affiliations:** 1 School of Information Environment, Tokyo Denki University, 2-1200 Muzai Gakuendai Inzai, Chiba, 270-1382, Japan; E-Mails: tmorotaka@gmail.com (K.T.); tsuruta@sie.dendai.ac.jp (S.T.); 2 Dipartimento di Tecnologie dell’Informazione, Universitàdegli Studi di Milano, Via Bramante 65, 26013 Crema (CR), Italy; E-Mails: marco.anisetti@unimi.it (M.A.); valerio.bellandi@unimi.it (V.B.); Paolo.Ceravolo@unimi.it (P.C.); ernesto.damiani@unimi.it (E.D.)

**Keywords:** context-based systems, sensor data interpretation, interpretation uncertainty

## Abstract

This paper proposes a methodology for sensor data interpretation that can combine sensor outputs with contexts represented as sets of annotated business rules. Sensor readings are interpreted to generate events labeled with the appropriate type and level of uncertainty. Then, the appropriate context is selected. Reconciliation of different uncertainty types is achieved by a simple technique that moves uncertainty from events to business rules by generating combs of standard Boolean predicates. Finally, context rules are evaluated together with the events to take a decision. The feasibility of our idea is demonstrated via a case study where a context-reasoning engine has been connected to simulated heartbeat sensors using prerecorded experimental data. We use sensor outputs to identify the proper context of operation of a system and trigger decision-making based on context information.

## 1. Introduction

Context-aware systems have the ability of adapting their behavior to their current operational context. Context changes are often triggered by events generated by sensors, like changes in location, time and communication involving people other than the user. Integration between sensors and context-based systems is being fostered by rapid evolution of technology; indeed, terminal devices like smart phones are now equipped with multiple sensors, such as video cameras or audio/video equipment, capable of collecting information from the environment. Our voice, hands, and whole body, monitored by sensors (e.g., of pressure or acceleration), are becoming the ultimate mobile input devices. The behavior of a software system may depend on where the user is located when a certain event takes place, where is she headed, or even whether is she sitting at her desk alone or walking accompanied by others.

A major problem in context-based systems is making sure that computers interpret sensor data correctly. Indeed, uncertainty in interpretation cannot be tackled simply by increasing sensor accuracy. It arises when alternative interpretations of sensor readings are possible, each carrying a certain amount of uncertainty. Let us consider a wrist accelerometer telling a software monitoring system that a person’s body is rapidly accelerating downward. While the acceleration can be measured very accurately, there is no way to decide with absolute certainty whether the person rearing the accelerometer is falling down, or is simply crashing on her bed or on her favorite sofa. Still, interpretation errors may bring a software system to making the wrong decision.

The contribution of this paper is twofold:

- A context description language based on an extension of the standard Semantics of Business Vocabulary and Business Rules (SBVR) (http://www.omg.org).
- A methodology for sensor data interpretation that can easily combine sensor outputs with human knowledge represented as SBVR rule sets.

In our approach, contexts are represented as sets of *annotated business rule*s [1] together with events generated by the interpretation of sensor readings. Each event comes labeled with the appropriate type and level of uncertainty. Events are also used to decide to switch context when appropriate. Active context’s rules are evaluated together with the events to make a decision. In order to carry out this evaluation, reconciliation of different uncertainty types is needed. It is achieved by a simple technique that moves uncertainty from events to the business rules by generating combs of standard Boolean predicates.

The paper is structured as follows: Section 2 surveys related work in the sensors and context-aware system domains, and Section 3 provides an outline of our approach. We deal with interpretation of sensor readings in Section 4, while in Section 5 we discuss our technique for uncertainty reconciliation. Section 6 describes the design of our context-based system, explaining via a case study how it works with simulated sensor data. In Section 7 we draw the conclusions and provide some outlook.

## 2. Related Work

Early sensor-based systems were based on the recognition of basic active modes, such as speech and handwriting, for which there is now a large body of research work. These systems sense and incorporate data about illumination, noise level, location, time, and people other than the user, as well as many other pieces of information to adjust their model of the user’s environment [2–4].

Facts representing users’ position and posture are highly dynamic; furthermore, their interpretation is hardly ever certain, especially within unsupervised environments. After some pioneering attempts [5], models for managing sensor data proposed have been more aimed at ensuring interoperability of the sensor infrastructure [6] than at reducing interpretation uncertainty. Still, there is an increasingly widespread consensus [7,8] that designers should be able to decide how to handle sensor data interpretation in order to improve understanding of the user’s intent.

Strategies for reducing interpretation uncertainty usually focus on the interface level, either by constraining user behavior to favor less error-prone interpretation (*i.e.*, “error reduction by design”), or by exploiting information coming from multiple sensors (*i.e.*, “error reduction by cross-modality”). However, users may not even be aware that their behavior is monitored by a system. They may also have a wrong understanding of what data the various devices capture, and how it is used. Traditional methods based on cross-modality error correction are also unfit to pervasive computing applications [9]. In this paper we use a different approach, proposing context-based techniques that do not require user involvement or even awareness [10].

Today, research on context is focusing on concepts like location, distance, movement and identity, especially in relation to mobile devices [11]. In this new setting, the active context plus sensor-generated events define *situations* where decisions must be timely taken.

In the sensor research community, the notion of context has been introduced for selecting active sensors based on contextual information and confidence [12]. Also, context information has been used as a way to keeping uncertainty under control when deploying sensors [13] or in sensor selection [14].

Several ontology-based approaches have been developed [6] for supporting this new generation of context-aware systems. e.g., by describing places, moments in time and their associated properties.

Other approaches have used context to model human reactions, especially in agent-based systems. Works in this area (see for instance the early survey [15] or the works [16,17]) are based on the assumption that expectations are implicit when a person or an agent is in a known context. Therefore, knowing a user’s context allows applying targeted procedures that lead to successfully manage events. When the situation changes, the agent must shift to a new context that best addresses the emerging situation. A related challenge is providing Web applications with access to the sensor (e.g., position or video) data flows generated by their users. Context data increasingly include information delivered by sensor devices: healthcare, homeland security, tactical training, *etc.* have a very real need to handle situations based on real-time information delivered by sensors [18]. A major reason why sensor interpretation is a difficult problem is that there are many types of uncertainty. Generally speaking, uncertainty arises when available knowledge is not sufficient to decide if a statement is true or false. Some researchers [19] have tried to classify uncertainty types. Depending on the problem at hand, a certain framework (such as fuzzy theories, probabilistic theories and possibility theory) can be more suitable than another. In our approach we explicitly represent the uncertainty type of each knowledge item inferred by interpreting sensor data, labeling each event with fuzzy or probabilistic uncertainty.

Once the uncertainty type is available for each sensor’s interpretation outcome, we face the problem of what to do with the uncertain assertions, *i.e.*, how to compose them. Unfortunately this is a difficult problem. Theoretical research has shown that one must exercise caution before conjoining assertions having different uncertainty models [20].

Some work in this direction imposed restrictions to the expressiveness of the logics. Among the most relevant studies, [21] proposes a definition of possibilistic fuzzy description logics by associating weights, representing degrees of uncertainty, to fuzzy description logic formulas (An extension of the Fuzzy Description Logics in the field of Possibility theory has been presented in [22]). Other works like [23,24] define probabilistic description logics programs by assigning probability degrees to fuzzy description logics programs.

In [22] the authors propose a framework for sharing information between three different models of uncertainty; in [25–27] the interoperability of uncertainty models has been studied and defined on a set of selected inference models. The *Ontology of Uncertainty* proposed by the W3C’s UR3W-XG incubator group [28] is a vocabulary to annotate different sources of information with different types of uncertainty. The URW3-XG incubator group was concerned with representation only, and did not specify how to deal with situations where more than one model is involved in the inference process; this is exactly the open issue we address in this paper. We are not aware of hybrid reasoning processes that can automatically handle flexible integration of different uncertainty types. Our choice of a semi-automatic technique is supported by recent results on automatic decision procedures suggesting that full automation of the composition of different decision procedures is an unlikely perspective [29].

## 3. Outline of the Approach

In our approach, each context is composed of *facts* or *events*, coming from the interpretation of sensor data, and a set of *rules*. Specific sets of meta-rules, called *transition rules*, specify under what conditions another context should be activated.

Our rule syntax is based on the notion of Business Rules (BRs). Business rules have been often used in the literature [30] as a starting point to develop business processes or as a way to validate them. Generally speaking, BRs define relevant domain entities, describe their properties/attributes and express the constraints on such properties that must hold in a given business situation.

Human modelers find it easy to express BRs in natural language, but some degree of formalization is necessary for automatic rule processing and interchanges. We formalize our business rules using the standard Specification on Semantics of Business Vocabulary and Business Rules (SBVR) (http://www.omg.org). The purpose of SBVR is to describe the semantics of a business model formally and without ambiguities. SBVR declarative format lends itself to easy translation into First Order Logic (FOL). For example, let us consider the following business rule expressed in natural language: “*For patient transfers, if the patient pick-up ward does not coincide with the drop-off one, the physician signing off the patient cannot be the same signing the patient in*”.

This rule can be parsed by a SBVR editor, obtaining an intermediate form “*For each referral X, if X.pickup.location ≠ X.dropoff.location, then X.pickup.physician ≠ X.dropoff.physician*”. In turn, the form can be readily translated into a FOL formula, like:

(1) ∀x∈R:(x.p.l≠x.d.l)→(x.p.p≠x.d.p)

We are now ready to explain some of our extensions to the SBVR business rules, focusing on those relevant for this paper. The interested reader will find a complete description of such extensions, including support for rule modality in [31]. Our extensions are aimed to represent contexts satisfying the three requirements of Section 1.

### 3.1. Dealing with Partial Knowledge

Next, we address two basic features of knowledge creation and sharing on the open web of sensors, *i.e.*, *(a) missing knowledge* (no agent performing an inference can be sure of holding all relevant information) and *(b) uncertainty* (sensor data interpretations have diverse uncertainty models).

Let us focus first on feature *(a)*, which is very important for some classes of applications. We do not want a service to rely on available information considering it complete, while in fact it is not. By avoiding to carry out inferences based on incomplete knowledge we also improve the system efficiency. Inferences based on incomplete knowledge often happen when information sources used by the service are not some trusted company databases, but the results of Web queries to foreign sites, which may be not up-to-date or simply unavailable. For each rule in our system, the designer can annotate each context’s rule set to decide if she wants it processed with Closed World (CW) or Open World assumption (OW) [32]. A CW assumption is the usual one for databases. In terms of our example, if a person name’s not in the list of chronic disease patients, the person will be considered as a non-chronic patient, regardless of how the list of chronic patients was put together. The OW assumption is introduced to prevent deriving negation from lack of knowledge. In terms of our example, under the OW assumption a person will be considered a chronic disease patient only if the list of chronic patients is provably complete. Note that choosing CW can lead to consequences for the hospital liability to claims (if a chronic patient dies or worsen his medical condition during an inter-ward transfer, insurance may not cover this case, and the hospital will remain liable). So, our context models are hybrid in nature, including both OW and CW contexts.

There are basically two ways to deploy a hybrid model [33]: (1) partitioning the rules and using multiple reasoners in parallel and (2) using a single reasoner, and adding meta-rules to handle the different reasoning types. The first technique is of course preferable when all the rules of each context follow either CW or OW: this way, when a context is active, its corresponding reasoning assumption (CW/OW) will be selected. In the rest of the paper, we will use the latter technique.

### 3.2. Sensor Interpretation Uncertainty

Our contexts are composed of rules and dynamically updated events from the interpretation of sensor data. For instance, a patient’s thermometer reading of *39C* (even without taking into account the uncertainty of the measurement) will translate into a “high fever” event with a fuzzy (*i.e.*, degree-based) uncertainty model and a value computable from standard tables. In other cases, sensor data-to-events mapping will involve probabilistic uncertainty. For instance, if a heartbeat sensor is mounted on an ambulance, the patient’s heartbeat can be easily grabbed and radio-transmitted ahead to the emergency ward; but the event of the patient being under stress will only be known with a degree of uncertainty whose nature is probabilistic but whose amount is unknown.

In this case, we use Bezier curves to estimate interpretation probabilities. The Bezier curves technique consists in asking human experts at which stake (for instance 1:10) they would be prepared to bet a small but not irrelevant amount of money (say, 100 Euro) on a given outcome, knowing the initial situation of the system. Their answer is then taken as a subjective estimation of the probability of that outcome. In the case of our example, we would ask a medical expert at which stake he would be prepared to bet 100 Euro that a senior citizen having a 110 heartbeat is under stress, and will assign the answer as probability. This procedure can be repeated for two or more points, to be later interpolated using low-degree Bezier curves. Such curves are then used to convert events into the probability of the system being in a given state. Such methods can support both evidence interpretation and context identification. Our system will guarantee that, given a 90% probability threshold for context identification, the system will only identify a context (e.g., the patient accident one) only if available information sources provide values that correspond to 90% probability via the current Bezier mapping.

In our system, we adopt an *accretion/resolution* (as opposed to a *synthesis/analysis*) approach [34,35]. In the *accretion/resolution* approach, the information received from sensors in the system will be stored as evidence without interpretation—a step called accretion. Then, when predicates based on events need to be evaluated, the stored evidence is examined and resolved to generate interpreted events. The advantage of this approach is that it saves the system from having to put effort into interpreting each sensor data item as it enters the system.

## 4. Interpretation of Sensor Data

Our aim is to handle interpretations with different uncertainty types. In terms of our running example, let us assume that access to a specific area of a hospital (e.g., a radiology room) is allowed to patients only if the physician in charge of their case accompanies them. To evaluate this condition the system relies on a single sensor, a video camera mounted on the door of the room. Although there is a single camera, two types of interpretation events are generated based on the video stream:

- Face recognition events: people within the camera scope are recognized and a message with their data (including their role) is sent to the inference system
- Location events: users’ distance from door is estimated, and a message is sent to the inference system if they are close to the door and facing it
- Relation events: if the data available in the hospital’s database say that two people are in a doctor-patient relation, a message is sent to the inference system

Note that, although all these events are generated with a level of interpretation uncertainty between 0 and 1, they involve two different uncertainty models: a probabilistic model for recognition (the face recognition software has a success rate less than 100%, depending on illumination conditions, occlusions, *etc.*), and an intensity-based model for the distance (the notion of “close” is fuzzy, as smaller distances are “closer”). Relation events are crisp: either the hospital database supports a relation or it does not, and the hospital database is assumed to be exhaustive (“CW assumption”, see Section 6). Our system accumulates events, and then evaluates the assertion to check if the door should be opened. The corresponding rule is given below (rule 2):

(2) Open(door1)←Patient(x)^Doctor(y)^nearTo(y,door1)^ nearTo(y,door1)^CareGiver(y,x).

The sensor interpretation software produces the following events (assertion 3). Each of them is labeled with its uncertainty type and uncertainty value.

(3) $$\begin{array}{l} \mathrm{event}1[\to ]\\ \to \mathrm{hasUncertainty}\;(\mathrm{event}1,0.8)\\ \to \mathrm{saidBy}\;(\mathrm{event}1,\mathrm{Driver}-\mathrm{camera}1)\\ \to \mathrm{saidAbout}\;(\mathrm{event}1,\mathrm{recognition})\\ \to \mathrm{contentOf}\;(\mathrm{event}1,\mathrm{PersonRecord}1)\\ \to \mathrm{uncertaintyModel}\;(\mathrm{event}1,\mathrm{Probability})\\ \to \mathrm{event}2[\to ]\\ \to \mathrm{hasUncertainty}\;(\mathrm{event}2,0.9)\\ \to \mathrm{saidBy}\;(\mathrm{event}2,\mathrm{Driver}-\mathrm{camera}1)\\ \to \mathrm{saidAbout}\;(\mathrm{event}2,\mathrm{distance})\\ \to \mathrm{contentOf}\;(\mathrm{event}2,\mathrm{PersoRecord}1)\\ \to \mathrm{uncertaintyModel}\;(\mathrm{event}2,\mathrm{fuzzy})\\ \mathrm{event}3[\to ]\\ \to \mathrm{hasCertainty}\;(\mathrm{event}3,0.8)\\ \to \mathrm{saidBy}\;(\mathrm{event}3,\mathrm{Driver}-\mathrm{camera}1)\\ \to \mathrm{saidAbout}\;(\mathrm{event}3,\mathrm{recognition})\\ \to \mathrm{contentOf}\;(\mathrm{event}3,\mathrm{PersonRecord}2)\\ \to \mathrm{uncertaintyModel}\;(\mathrm{event}3,\mathrm{Probability})\\ \mathrm{event}4[\to ]\\ \to \mathrm{hasCertainty}\;(\mathrm{event}4,0.9)\\ \to \mathrm{saidBy}\;(\mathrm{event}4,\mathrm{Driver}-\mathrm{camera}1)\\ \to \mathrm{saidAbout}\;(\mathrm{event}4,\mathrm{distance})\\ \to \mathrm{contentOf}\;(\mathrm{event}4,\mathrm{PersonRecord}2)\\ \to \mathrm{uncertaintyModel}\;(\mathrm{event}4,\mathrm{fuzzy})\\ \mathrm{event}5[\to ]\\ \to \mathrm{hasCertainty}\;(\mathrm{event}5,1)\\ \to \mathrm{saidBy}\;(\mathrm{event}5,\mathrm{NHDB})\\ \to \mathrm{saidAbout}\;(\mathrm{event}5,\mathrm{relation})\\ \to \mathrm{contentOf}\;(\mathrm{event}5,\mathrm{CareGiver}\;(\mathrm{PersonRecord}1,\mathrm{PersonRecord}2))\\ \to \mathrm{uncertaintyModel}\;(\mathrm{event}5,\mathrm{None}) \end{array}$$

Now, explicit annotation of uncertainty types can help the system to compute conjunction consistently. Events 1 and 3 are conjoined probabilistically, *i.e.*, taking the union of the probabilities:

p(event1^event3)=p(event1)∩p(event3)=0.8*0.9=0.72

Events 2 and 4 are conjoined fuzzily, *i.e.*, taking the minimum:

p(event2^event4)=min(p(event2),p(event4))=min(0.8,0.9)=0.8

Note that Event 5 is regarded as absolutely certain (certainty = 1) because is not generated by a sensor but is obtained by querying the hospital database. Of course, uncertainty for this source can exist, but it is not related to vagueness as in the previous examples but to incompleteness (the relation may exist but not be shown in the database) or expiration (the relation may no longer exist but the database records still show it). We shall deal with incompleteness in Section 6. At the final step the systems would need to conjoin *P*_1_ = *p*(*event* 1 ∧ *event* 3), *P*_2_ = *p*(*event* 3 ∧ *event* 4) and of course *P*_3_ = *p*(*event* 5), the latter being 1. Although frequently done in real systems, this conjunction (*P*_1_ ∧ *P*_2_ ∧ *P*_3_) is arbitrary, as uncertainty types of the operands do not match. Indeed, computing a threshold on the result of a badly chosen final conjunction (say, a weighted average), would allow an intruder looking reasonably like one of the doctors to compensate for a low probability of recognition by putting himself closer to the door!

In the next Section 5, we shall avoid this undesired compensation by taking separate thresholds for assertions having different uncertainty types, and then conjoining the resulting standard Boolean predicates.

## 5. Sensor Uncertainty Reduction

The simple example of Section 4 involved some manual intervention in the choice of the individual uncertainty models and, perhaps more importantly, of the final conjunction. One could hope to use some meta-reasoning engine for automatically selecting a suitable conjunction; however, generally speaking, automatically composing decision procedures involving different uncertainty models is still an open problem, and choosing a conjunction to avoid undesired compensation effects is more an art than a science [36,37]. Past research has shown that a combination of a rule-based approach with probability theory can only be done at the price of inconsistencies [22] especially if event independence cannot be assumed safely. In order to avoid having to conjoin assertions having heterogeneous uncertainty model, we define a family of crisp (*i.e.*, ordinary) predicates [38]. Each predicate evaluates to true for the value of uncertainty (probability, fuzzy degree *etc.*) that would bring the modeler to assert it with the confidence hard-wired in its names.

For example let us consider the predicate *WithConfidenceGreaterEqual2/3*() (for brevity, *WCGE2/3*()). It is a crisp predicate; the context modeler defines the single argument (in the [0.1] interval) that makes it true. To fix our mind, let us assume the modeler chooses *WCGE2/3*(0.8) = true. The modeler is stating that an uncertainty value of 0.8 provides a subjective vote of confidence of 2/3. This definition should be repeated for each uncertain predicate in a SBVR rule. With reference to the example of Section 4.1, we can then restate rule 2 as follows:

(4) Open(door1)←WCGE9/10(Patient(x))^WCGE9/10(Doctor(y))^WCGE8/10(nearTo(y,door1))^WCGE8/10(nearTo(y,door1))^WCGE1(CareGiver(y,x)).

Note that there must be a different *WCGE* predicate for each uncertain predicate in the original condition. To avoid cumbersome indexing we introduce an abuse of notation using the name of the original uncertain predicate as an argument of the *WCGE* protocol. Nevertheless we are still using the same *WCGE* for predicates like *nearTo*(*y*, *door1*) and *nearTo*(*x*, *door1*) having the same uncertainty type. This is often undesirable; in this case, for instance, we might want to allow that if one of the pair is surely identified this compensates for a slightly lower probability in the identification of the other. We could therefore define a different *WCGE* for each signature of the predicates in the original formula.

This rule can be evaluated straightforwardly, because all the predicates in its antecedent are Boolean; no compensation can take place among different uncertainty models. We shall discuss the details of this construction in Section 7.

## 6. Design and Implementation of the Context Based System

**Figure 1.**
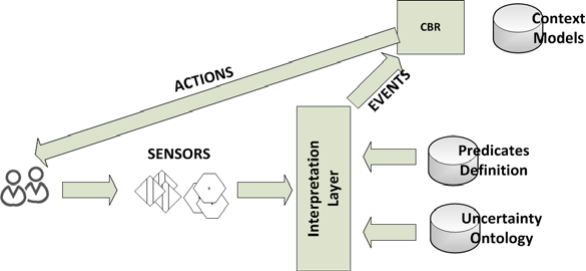
Architecture.

We are now ready to show how our contexts, composed of annotated business rules and of events that come from the interpretation of sensor data, can be fed into an efficient context-based reasoning system. As stated in Section 1, we shall adopt the rule-based context representation of the CxBR environment. Figure 1 shows our architecture.

### 6.1. Structuring the Context-Based Model

**Figure 2.**
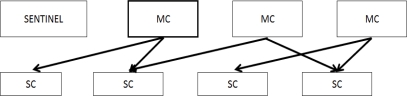
Our context Model. The box with thick borders is the default MC.

The CxBR environment is based on the idea that a situation calls for a set of actions that properly addresses it. A transition to another set of actions and procedures may be required to address new situations. In general, things likely to happen under a given situation are limited and well known. CxBR encapsulates knowledge about appropriate actions for specific situations into hierarchically organized contexts. All the behavioral knowledge is stored in a database of Context Models (*i.e.*, the collection of all contexts). Figure 2 shows our context models.

The top layer of contexts is the one Major Contexts (MC) (including the default when the system starts) and then layers of Minor Contexts (e.g., Sub-Contexts (*SC*), Sub-Sub-Contexts, (*SSC*) and so on). MCs contain *context rules*, functions that implement actions, and an asynchronously activated *sentinel context* used to enable context switch among MCs. Identification of a new context is simple, because only a limited subset of all contexts can be activated under the currently active context. SCs encapsulate blocks of rules and functions performed by MCs to reduce complexity.

Using the technique presented in Section 4, we can avoid dealing with vagueness and imprecision at this level; all rules in CxBR contexts are crisp. Still, the CxBR must handle the CW/OW annotations.

The CxBR context structure for our hospital door example has two MCs, NORMAL (the default context) and EMERGENCY. NORMAL and all its sub-contexts are handled under CW assumption, *i.e.*, assuming that the information currently held by the service is exhaustive. Namely, the system can derive negation by lack of information, as this will not have dire consequences in this context. EMERGENCY and its sub-contexts are handled with a more cautious OW assumption (namely, it is not applied to derive negation from lack of information). An additional context, called *sentinel*, uses incoming events (sensor data interpretations) to periodically re-compute predicates that that may trigger *transition rules* that cause a context switch.

### 6.2. Case Study: Monitoring Critical Patients

**Figure 3.**
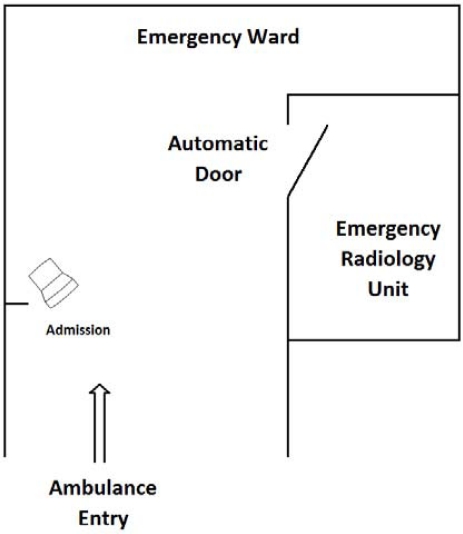
Case Study scenario.

In intensive care units the use of diagnostic devices must take into account criticality of the patients. In our case study (Figure 3) access to a diagnostic device is controlled by different rule sets depending on the active contexts.

In our case study, a physician or a paramedic positions a heartbeat sensor on the patient’s body. Our interpretation rule interprets the heartbeat value to infer a critical condition event with fuzzy uncertainty type. The technique outlined Section 5 is then used, applying a comb of Boolean predicates of the type *isCriticalX/Y* to generate events to be used in the context-based reasoning phase. During NORMAL operation, the automatic door to the emergency radiology room can only be opened when the physician in charge and one of her patient are both present (rule 2). Suppose now that a message notifying the imminent arrival of a patient with a heart attack reaches the hospital. In this case, the SENTINEL context evaluates its context-switching rule and switches to the EMERGENCY context. The rules are listed below:

SENTINEL: Minor Context:

*Action rules:*

(5) readSensor():IsCritical(x)←isCritical9/10(x.heartbeat)

NORMAL: Major Context: CW, P

*Transition rules:*

(6) SwitchTo(EMERGENCY)←IsCritical(x)

*Action rules:*

(7) P:Open(door1)←Patient(x)^Doctor(y)^nearTo(x,door1)^nearTo(y,door1)^CareGiver(y,x).

EMERGENCY: Major Context: OW, O

*Action rules:*

(8) P:Open(door1)←Patient(x)^Doctor(y)^nearTo(x,door1)^nearTo(y,door1)^CareGiver(y,x).O:CallSecurity()←Open(door1)

Let us consider how the CW/OW assumption described in the previous Sections controls the operation of our context-based reasoning engine.

- CW/OW annotations are compiled into a meta-predicate specifying when lack of knowledge (*CareGiver*(*x*, *y*) = *NA*) must be treated as negation. When in the NORMAL context, the system works under the CW assumption. So, if a patient is coming in and the hospital database is unavailable from the admission terminal (Figure 2), *CareGiver*(*x*, *y*) times out and the door stays close (in a way, the system derives negation from lack of information). Once we are in the EMERGENCY context, the system will be able to derive *Open(door1)* even if the hospital database is not online. Of course the actual heartbeat frequency may not trigger the predicate *isCritical9/10* required to switching context. If another predicate of the family, say *isCritical7/10*, is selected, the context switch will NOT take place and the context-based system will stay in the NORMAL operational context.

### 6.3. Experimentation

Our experimentation took in account a simple setting, where a heartbeat sensor is used. Many noninvasive methods exist for electronically sensing the human heartbeat. They can be based on acoustic (stethoscope or Doppler), mechanic (sphygmomanometer), electric (EKG), and optical signals. The optical technique exploits the fact that tiny subcutaneous blood vessels in any patch of skin (e.g., the fingertips) furnished with a good blood supply, alternately expand and contract in time with the heartbeat. An ordinary infrared LED/phototransistor pair can sense this rhythmic change as small but detectable variations in skin contrast. This type of sensor is really non-invasive and easy to place over a patient even in a critical situation. An example of such a low-cost optical heart-rate sensor is the one used with Arduino platform [39]. The principle of this sensor is that an optical heart-rate pulse sensor, light is shot into a fingertip or ear lobe. The light either bounces back to a light sensor, or gets absorbed by blood cells.

Heartbeat data have been used for different kind of analysis. In [40] the classification performance and generalization were studied using publicly available databases: the MIT-BIH Arrhythmia, the MIT-BIH Supraventricular Arrhythmia, and the St. Petersburg Institute of Cardiological Technics (INCART) databases. However, these databases are all ECG databases, while for the case study of this paper we are focusing on the heartbeat rate only. For our experimentation we collected data from our sensor and expressed it as series of consecutive heartbeat rate. For testing extreme condition with very low heartbeat rate or very high heartbeat rate, we introduce some *ad hoc* sequences of critical heartbeat rate. We also use manually constructed heartbeat rate streams for covering the entire heartbeat rate domain.

Our experimentation set up is based on Matlab 7.10 on top of Intel Core i-7 machine with 4GB of RAM. The evaluation time is the CPU time measured with Matlab and is referred to the average CPU time of 1,000 evaluation. The average mitigates the fluctuation in CPU time due to other processes running in the system.

**Figure 4.**
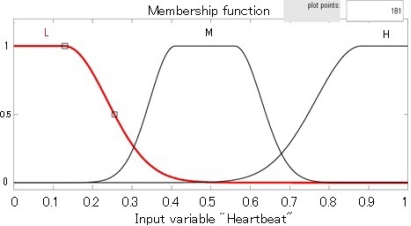
Fuzzy Membership for predicate *isCritical().* Values on the Heartbeat axis are normalized between 0 and 150 heartbeat rate.

**Figure 5.**
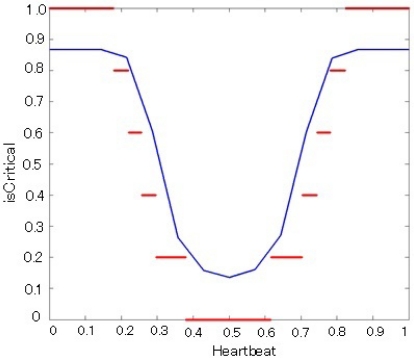
The output surface for the fuzzy *isCritical()* predicate in blue and the output surface for the crisp *isCritical()* predicate in red.

Table 1 shows some results of the evaluation of the crisp version of *isCritical()* predicates for the SENTINEL context. In order to assess the performance of our approach, we compare our results to a benchmark: a standard fuzzy controller based on a fuzzy version of the *isCritical()* predicate (Figure 4). Figure 5 shows the decision surface of fuzzy controller in blue and the decision surface of our crisp based controller in red. The simple fuzzy rules used for the fuzzy controller are the following: *if (Heartbeat is L) then (isCritical is H); if (Heartbeat is H) then (isCritical is H); if (Heartbeat is M) then (isCritical is L)*.

As shown in Figure 5, our experimentation considered a single fuzzy membership function that was translated into a comb of our WCGE crisp predicates. Each fuzzy rule was then translated into one or more crisp rules using the predicates from the comb. The resulting crisp rules were used to handle the same inputs and the benchmark fuzzy controller. Table 1 shows how our approach mimics well the behavior of the fuzzy controller, with a much lower processing time. This way, interpretation overhead is kept to a minimum. Note, however, the stepwise behavior of our technique: the Boolean predicate *isCritical9/10* is not triggered when heartbeat equals to 30, while it is triggered in the case of 27. This suggests that the interval between predicates of the comb must be selected carefully in order to smooth threshold cuts. Generally speaking, the comb intervals can be also selected *ad hoc*, depending on the application and the uncertainty type. For fuzzy uncertainty, we use a simple heuristics: the interval between two crisp predicates *P_i_* and *P_i+1_* in the comb is inversely proportional to the derivative of the membership function at the point where *P_i_* *= true*. This heuristics ensures that the highest the derivative, the closer the intervals.

### 6.4. The Engine

Once uncertainty has been reconciled using the technique described above, fast computation of context rules becomes essential. We developed a parser that receives context representations from our SBVR editor and compiles FNL rules into a flat XML encoding of First-Order-Logics (FOL) that can be used for reasoning. Then, we feed this XML context base to an efficient Context-based Reasoning (CxBR) engine. CxBR ([17,41]), entirely written in Java, was originally designed to efficiently control time-based missions. Its ability to efficiently and effectively control agent platforms in tactical situations has been proven in several application areas such as automobile driving [41], submarine warfare [16], project management [41], *etc.*

**Figure 6.**
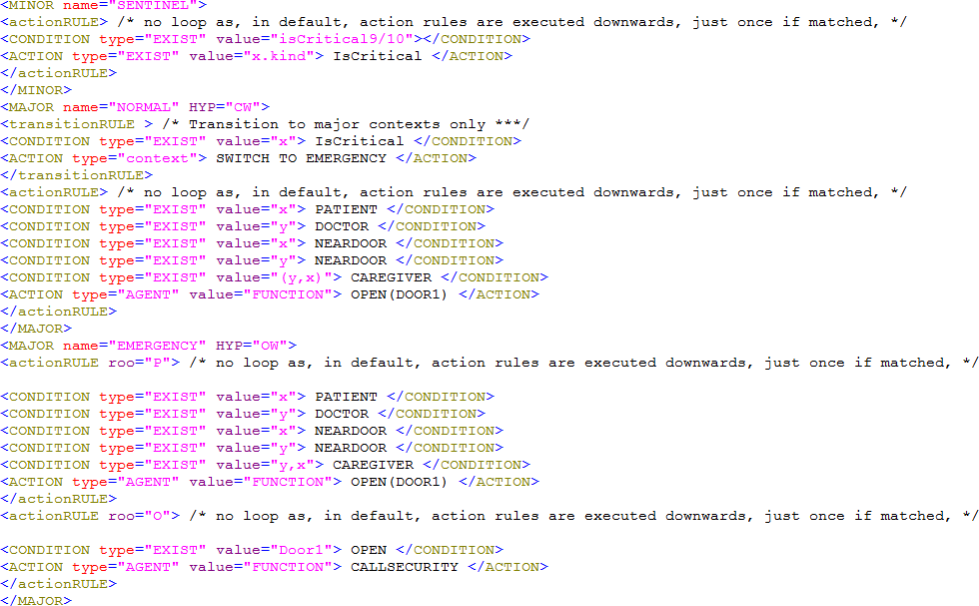
XML incarnation of the CxBR context base.

In the example of Figure 6, <CONDITION> tags check the system’s facts base while <ACTION> ones update it based on the condition’s outcome. The facts base is updated by (the interpretation) of the sensors streams, in our case the heartbeat sensor channel. Our reasoning algorithm ensures efficiency through no backtracking, as follows:

- Step 1. Default context (NORMAL) is activated.
- Step 2. Until end-of-mission criteria is reached, do:
  - (a) If mission goals attained · go to step 3
  - (b) If other end-of-mission criteria reached · go to step 4.
  - (c) Premises of all Transition rules are evaluated to determine whether the situation has changed enough to transit to a new MC. Thus, the control jumps among MCs. Default transition list of step 1 is checked at the last stage of this evaluation as default, usually transiting to Normal MC. If change in situation exists:
    * De-activate the currently active MC.
    * Execute Transition to activate the appropriate new MC.
    * Initialize the new active MC including the followings:
    * If OW is specified, set the value of OW in fact base to True, else reset to False.
    * Return to step 2(a)
  - (d) Premises of all Action rules are evaluated to determine whether any action or any minor context is to be activated. Other major contexts or lower levels of minor contexts can be activated. Thus, neither backtracking nor loop occurs among sub-contexts. If any action be activated:
    * execute action parts, including control functions or auxiliary agents (AAs), passing them OW value and the prioritized modality strategy
    * return to step 2(d)
    * If any minor context be activated, Action rules are fired to activate it (this activation does not deactivate the activate Major Context)
    * Initialize minor context including the followings:
      · If OW is specified, set OW value in fact base to True,
      · if CW is specified, set OW value to False
  - (e) Return to step 2(a)
- Step 3. Mission successfully completed
- Step 4. Stop.

As shown in step 2(c), control jumps freely among CxBR’s Major Contexts to cope with various kinds of situations or events. However, our engine traverses contexts only downward as shown in step 2(d). Therefore, neither any backtracks nor any loops occur among minor contests and to their own major contests. Furthermore, as shown in step 2(c) and step 2(d), setting the value of OW is done automatically, according to the annotation such as *OW/CW*.

## 7. Conclusions

We have described a novel approach to context modeling based on an expressive context definition language based on SBVR, showing how it can be interfaced with sensors by generating events from the interpretation of sensor readings. Although mathematical models for reasoning with uncertain information have been successfully applied in several situations, interpretation of sensor data exhibit must deal with different types of uncertainty. Our approach allows for reconciling different types of uncertainty and showed a simple case study in the healthcare domain involving fuzzy uncertainty. There is in our opinion little doubt that the next generation of Web applications will need to handle much more complex and subtle context knowledge than current systems. We plan to apply our approach to the problem of interpreting the user behavior and level of awareness the key domain of computer-assisted collaboration and interactive e-learning systems [42].

## Figures and Tables

**Table 1.** Experimental results. “N” and “E” stand for “NORMAL” and EMERGENCY contexts.

**Heartbeat**	**Activated crisp rules**	**Fuzzy value**	**MC**	**Crisp eval. time**	**Fuzzy eval. time**
75	isCritical0/10	0.1358	N	0.000014 s	0.000813 s
95	isCritical2/10	0.2416	N	0.000015 s	0.00075 s
130	isCritical9/10 isCritical8/10 ….	0.8673	E	0.000018 s	0.00074 s
30	isCritical8/10 isCritical7/10 ….	0.8547	N	0.000024 s	0.00072 s
27	isCritical9/10 isCritical8/10 …	0.8629	E	0.000018 s	0.00076 s
